# Work changes and individual, cancer‐related, and work‐related predictors of decreased work participation among African American cancer survivors

**DOI:** 10.1002/cam4.3512

**Published:** 2020-11-07

**Authors:** Theresa A. Hastert, Anne C. Kirchhoff, Matthew P. Banegas, Joanna F. Morales, Mrudula Nair, Jennifer L. Beebe‐Dimmer, Stephanie S. Pandolfi, Tara E. Baird, Ann G. Schwartz

**Affiliations:** ^1^ Wayne State University School of Medicine Detroit MI USA; ^2^ Karmanos Cancer Institute Detroit MI USA; ^3^ Huntsman Cancer Institute University of Utah Salt Lake City UT USA; ^4^ Kaiser Permanente Center for Health Research Portland OR USA; ^5^ Triage Cancer Chicago IL USA

**Keywords:** African American, cancer survivors, employment

## Abstract

African American cancer survivors disproportionately experience financial difficulties after cancer. Decreased work participation (going from being employed full time to part time or from employed to not employed) can contribute to financial hardship after cancer but employment outcomes among African American cancer survivors have not been well described. This study estimates the prevalence of work changes and identifies factors associated with decreased work participation among African American cancer survivors. We analyzed data from 916 African American breast, colorectal, lung, and prostate cancer survivors who participated in the Detroit Research on Cancer Survivors (ROCS) cohort and were employed before their cancer diagnosis. Modified Poisson models estimated prevalence ratios of decreased work participation and work changes, including changes to hours, duties, or schedules, between diagnosis and ROCS enrollment controlling for sociodemographic and cancer‐related factors. Nearly half of employed survivors made changes to their schedules, duties, or hours worked due to cancer and 34.6% took at least one month off of work, including 18% who took at least one month of unpaid time off. More survivors employed full time (vs. part time) at diagnosis were on disability at ROCS enrollment (18.7% vs. 12.6%, *P* < 0.001), while fewer were unemployed (5.9% vs. 15.7%, *P* < 0.001). Nearly half (47.5%) of employed survivors decreased work participation. Taking paid time off was not associated with decreased work participation; however, taking unpaid time off and making work changes were associated with prevalence ratios of decreased work participation of 1.29 (95% CI: 1.03, 1.62) and 1.37 (95% CI: 1.07, 1.75), respectively. Employment disruptions are common after a cancer diagnosis. Survivors who take unpaid time off and make other work changes may be particularly vulnerable to experiencing decreased work participation.

## Graphical abstract

Nearly half of employed African American cancer survivors decreased work participation (went from full time to part time or employed to not employed) after cancer diagnosis. Taking unpaid (but not paid) time off and changing work hours, duties, and/or schedules were associated with decreased work participation.

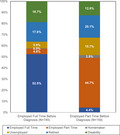

## 1 INTRODUCTION

Nearly half of cancer survivors report adverse financial outcomes or even “catastrophic” financial hardship related to cancer or cancer treatment.^1^, ^2^, ^3^ Financial hardship due to cancer is associated with lower health‐related quality of life (HRQOL),^2^ lower treatment adherence,^3^ and even with higher mortality.^4^

Difficulty maintaining employment during and returning to employment after cancer treatment represents a potentially modifiable risk factor for financial hardship among cancer survivors. Working‐age cancer survivors are more likely to be unemployed than healthy controls.^5^ Cancer survivors can miss weeks or months of work due to cancer treatment, and individual and family earnings can drop substantially, promoting and exacerbating financial hardship after cancer.^6^ By some estimates, nearly 40% of working adults diagnosed with cancer do not return to work within 1 year.^7^, ^8^ Many cancer survivors also make changes to their hours, duties, or work schedules related to cancer and cancer treatment,^9^, ^10^ and evidence suggests that survivors with accommodating employers are more likely to return to work.^11^, ^12^

Understanding the potential impact of cancer and cancer treatment among African American cancer survivors is particularly important. African Americans are underrepresented in white collar and professional occupations^13^, ^14^ that have the best job retention after cancer, and are overrepresented in positions requiring physically demanding work that is associated with reduced employment after cancer.^15^, ^16^, ^17^, ^18^, ^19^ Decreased employment^12^, ^19^, ^20^ and financial hardship^2^, ^3^, ^21^ are more common among African American than white survivors.

Investigators have identified the need for research designed to better understand employment outcomes among racial and ethnic minority cancer patients;^22^ however, few previous studies have included large numbers of African American survivors. The purpose of this study is to describe the prevalence of work changes and decreased work participation and to identify individual, cancer‐related, and work‐related predictors of decreased employment in a population‐based cohort of African American cancer survivors.

## 2 METHODS

### 2.1 Study population

Detroit Research on Cancer Survivors (ROCS) is a population‐based cohort designed to investigate associations between medical history, health behaviors, financial factors, and health‐related outcomes among African American cancer survivors.^23^, ^24^ Survivors were eligible to join the cohort if they were diagnosed with primary, invasive breast, colorectal, lung, or prostate cancer since 1 January 2013 and identified through the Metropolitan Detroit Cancer Surveillance System (MDCSS), a population‐based cancer registry covering metropolitan Detroit and a founding participant in NCI’s Surveillance, Epidemiology and End Results (SEER) program. The Wayne State University Institutional Review Board approved this research.

Results include cross‐sectional data from the first 2500 ROCS respondents to the ROCS enrollment survey. We excluded responses from 653 participants who completed an early version of the survey without the employment impact questions, and 931 participants who were not employed full time or part time either before diagnosis or at the time of ROCS enrollment, leaving an analysis sample of N = 916.

### 2.2 Study measures

Participants were asked two questions related to employment status, “Which of the following phrases best describes your current employment status?” and “What was your employment status prior to your cancer diagnosis?” Options for both included: employed full time (including self‐employed), employed part time (including self‐employed), homemaker, unemployed, retired, on disability, and an option for participants to write in another response.

*Decreased work participation* was calculated using participants’ self‐reported current employment status at ROCS baseline and prior to cancer diagnosis. Survivors were considered to have decreased work participation if they went from being employed full or part time to not employed (homemaker, unemployed, on disability) or employed full time to employed part time between diagnosis and ROCS baseline.

*Participant characteristics* include age at diagnosis (mean: 55.9, range: 27–79) obtained from MDCSS, as well as self‐reported sex (male, female), educational attainment (less than high school, high school/GED, some college or technical school/2‐year degree, 4‐year college degree, graduate school), marital status (married, living with partner in a marriage‐like relationship, widowed, divorced, separated, never married), household income (<$20,000, $20,000–39,999, $40,000–59,999, $60,000–79,999, $80,000+), and usual occupation using categories presented in Table 1. Participant characteristics reflect information at the time of ROCS baseline survey.

**TABLE 1:** Participant characteristics

	Total	%
	N = 916	
Survivor characteristics		
Sex		
Female	483	52.7
Male	433	47.3
Age at diagnosis		
<55	368	40.2
55–64	397	43.3
65+	151	16.5
Age (mean, SD)	58.2	9.4
Education		
High school or below	263	29.1
Some college	361	40.0
College graduate	279	30.9
Marital status		
Married/living with partner	400	43.9
Widowed	55	6.0
Divorced/separated	230	25.3
Never married	226	24.8
Annual household income		
Less than $20,000	256	29.9
$20,000–39,999	194	22.6
$40,000–79,999	577	28.5
$80,000+	163	19.0
Usual occupation		
Professional/technical	256	28.0
Manager/administrator	109	11.9
Clerical	72	7.9
Service	145	15.8
Craftsperson	77	8.4
Operative	171	18.7
Other/missing	86	9.4
Cancer‐related factors		
Site		
Breast	371	40.5
Colorectal	132	14.4
Lung	72	7.9
Prostate	341	37.2
Stage		
Localized	552	60.5
Regional	284	31.1
Distant	76	8.3
Treatments received		
Surgery	231	25.4
Radiation	463	50.9
Chemotherapy	525	57.7
Currently in treatment for initial cancer diagnosis		
No	692	76.8
Yes	209	23.2
Months since diagnosis		
<18	410	44.8
18–23	75	8.2
24+	431	47.1
Months since diagnosis (mean, SD)	27.0	19.6

Abbreviations: SD, standard deviation.

*Cancer*‐*related factors* include site, SEER summary stage, and time since diagnosis as well as participant reports of treatments received (any surgery, chemotherapy, radiation), and whether they completed treatment for their initial cancer diagnosis.

*Work*‐*related factors* were measured by asking employed survivors whether, since their cancer diagnosis, they had to do any of the following (each *yes*/*no*): change their work schedule, change the number of hours worked each week, change job duties, take extended paid time off from work, take unpaid time off from work. Participants were counted as having made work changes if they answered “yes” to any of these questions. Survivors who indicated they took extended paid or unpaid time off were then asked the length of time taken off (<*1* *week*, *1* *week* *to* *1* *month*, *1*–*3* *months*, *3*–*6* *months*, *6* *months* *or* *more*) using questions adapted from the Medical Expenditure Panel Survey (MEPS): Experiences with Cancer Survivorship Supplement.^25^

### 2.3 Statistical analysis

All analyses were conducted in Stata/SE 16. We used modified Poisson regression with robust standard errors to estimate prevalence ratios and 95% confidence intervals for decreased work participation by survivor, cancer‐related, and work‐related factors.^26^, ^27^ Models controlling for sociodemographic characteristics included survivor sex, age at diagnosis, education, marital status, household income, and occupation using categories presented in Table 1. Models controlling for cancer‐related factors included cancer site, stage, treatments received, treatment status, and time since diagnosis using categories in Table 1. Because it is not clear whether being employed before cancer diagnosis and retired at ROCS enrollment was due to cancer or represented an adverse financial outcome, models of decreased work participation excluded participants who reported being retired at ROCS enrollment. Supplementary tables provide results including being retired at ROCS enrollment as a form of decreased employment.

## 3 RESULTS

Table 1 presents sociodemographic and cancer‐related characteristics of cancer survivors employed before their cancer diagnosis. Mean age at diagnosis was 55.9 years and mean age at survey completion was 58.2 years. More than half of participants were women, and a plurality were married or living with a partner. Approximately 30% completed college. The largest proportion of participants reported incomes below $20,000 while the fewest reported incomes of $80,000 or more. Professional or technical occupations were reported most frequently, followed by operative, service, and management/administration.

Breast was the most common cancer, followed by prostate, colorectal, and lung. Approximately 60% were diagnosed with local disease. An average of 27 (median 21, range 2–76) months elapsed between diagnosis and ROCS survey.

Prevalence of work‐related outcomes is given in Table 2. More than one‐third of survivors took at least one month off of work. Nearly one quarter reported taking extended paid time off, including 21% who took at least one month of paid sick time and 9% who took at least one month of paid vacation. One‐third took unpaid time off, including 18% who took at least one month of unpaid time off. Nearly half of survivors made one or more other work changes, including 24% who changed their duties, 43% who changed their schedule, and 38% who changed the number of hours worked each week.

**TABLE 2:** Prevalence of extended time off and work changes since cancer diagnosis

	N	%
Took at least 1 month off (paid or unpaid)	266	34.6
Extended paid time off from work	184	23.7
1+ month sick time	161	21.1
1+ month vacation time	68	8.9
Any unpaid time off	262	33.7
1+ month unpaid time	142	18.4
Any work change	379	48.5
Changed work duties	184	23.8
Changed hours worked each week	297	38.4
Changed work schedule since diagnosis	335	43.0

**FIGURE 1:**
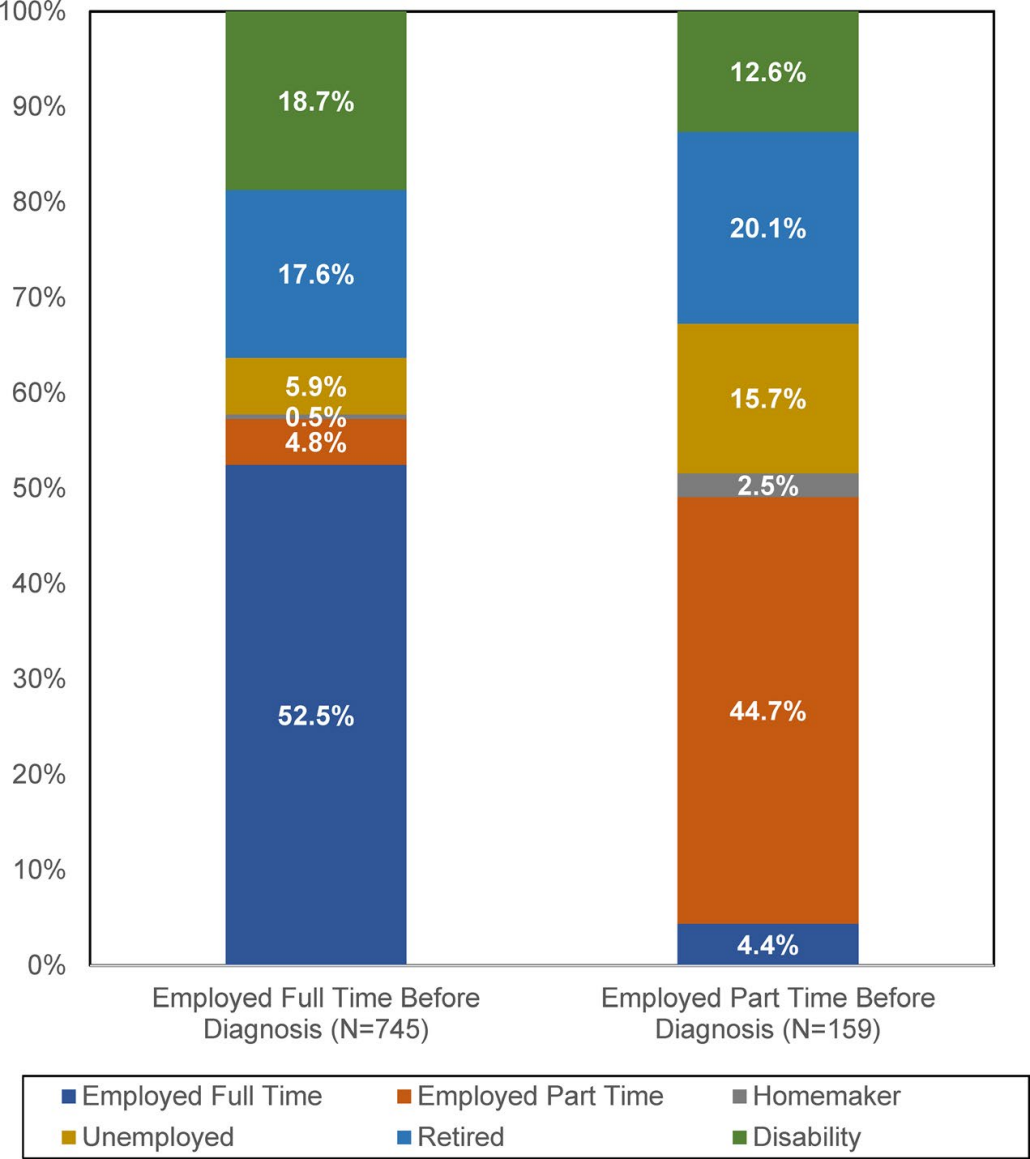
Survivor employment status at ROCS baseline by pre‐diagnosis employment status

Figure 1 gives the employment status at ROCS enrollment of survivors who reported being employed full or part time before their cancer diagnosis. Among survivors working full time before diagnosis, 52.5% reported full time employment at ROCS enrollment, whereas 4.8% went to part‐time work. Among survivors working part time before diagnosis, 4.4% changed to full time employment by ROCS enrollment and 44.7% remained employed part time. Being on disability at ROCS enrollment was less common among survivors employed part time before diagnosis compared to those employed full time (12.6% vs. 18.7%, *P* < 0.001), while part time workers were nearly three times as likely to be unemployed at ROCS enrollment (15.7% vs. 5.9%, *P* < 0.001).

Predictors of decreased work participation, excluding retirement, are presented in Table 3. In models controlling for survivor characteristics, sex and age were not associated with decreased work participation; however, decreased work participation was less common among never‐married survivors compared to those who were married or living with a partner. Household income was inversely associated with decreased work participation. Decreased work participation was more common among survivors reporting lower levels of educational attainment and among craftspersons and those in service and operative occupations compared with professional or technical occupations in unadjusted models, but not adjusted models.

**TABLE 3:** Decreased employment participation by participant sociodemographic, cancer‐related, and work‐related characteristics

	Decreased employment participation		Unadjusted		Adjusted^a^	
	N	%	PR	95% CI	PR	95% CI
Survivor characteristics	272	36.1				
Sex						
Female	151	36.7	1.00	Ref	1.00	Ref
Male	121	35.4	0.96	(0.80, 1.17)	0.93	(0.67, 1.29)
Age at diagnosis						
<55	118	33.2	1.00	Ref	1.00	Ref
55–64	134	41.6	1.26	(1.03, 1.53)	1.20	(0.98, 1.46)
65+	20	26.7	0.80	(0.54, 1.20)	0.91	(0.63, 1.33)
Education						
High school or below	116	53.2	1.00	Ref	1.00	Ref
Some college	110	37.4	0.70	(0.58, 0.85)	0.94	(0.77, 1.14)
College graduate	41	17.8	0.33	(0.25, 0.45)	0.75	(0.53, 1.07)
Marital status						
Married/living with partner	96	29.7	1.00	Ref	1.00	Ref
Widowed	16	50.0	1.68	(1.14, 2.47)	0.73	(0.52, 1.04)
Divorced/separated	73	39.0	1.31	(1.03, 1.68)	0.82	(0.65, 1.02)
Never married	86	41.6	1.40	(1.10, 1.76)	0.71	(0.56, 0.89)
Annual household income						
<$20,000	147	70.0	1.00	Ref	1.00	Ref
$20,000–39,999	56	35.7	0.51	(0.41, 0.64)	0.53	(0.42, 0.67)
$40,000–79,999	31	15.7	0.22	(0.16, 0.31)	0.23	(0.16, 0.33)
$80,000+	16	11.1	0.16	(0.10, 0.25)	0.17	(0.10, 0.29)
Usual occupation						
Professional/technical	56	26.5	1.00	Ref	1.00	Ref
Manager/administrator	22	25.0	0.94	(0.62, 1.44)	1.15	(0.80, 1.65)
Clerical	17	28.3	1.07	(0.67, 1.69)	0.75	(0.45, 1.23)
Service	67	53.2	2.00	(1.52, 2.65)	0.85	(0.63, 1.15)
Craftsperson	25	41.7	1.57	(1.08, 2.28)	0.87	(0.58, 1.30)
Operative	54	39.1	1.67	(1.18, 2.36)	0.93	(0.68, 1.28)
Cancer‐related factors						
Site						
Breast	101	31.6	1.00	ref	1.00	ref
Colorectal	58	51.8	1.64	(1.29, 2.09)	1.50	(1.07, 2.12)
Lung	31	67.4	2.14	(1.65, 2.76)	1.65	(1.19, 2.27)
Prostate	82	29.8	0.94	(0.74, 1.20)	1.23	(0.80, 1.88)
Stage						
Localized	129	28.5	1.00	ref	1.00	ref
Regional	96	41.0	1.44	(1.17, 1.78)	1.10	(0.89, 1.36)
Distant	46	73.0	2.56	(2.08, 3.16)	1.37	(1.02, 1.84)
Treatment						
Any surgery (vs. none)	200	35.2	0.90	(0.73, 1.11)	1.07	(0.85, 1.35)
Any chemotherapy (vs. none)	144	44.7	1.52	(1.26, 1.84)	1.28	(1.00, 1.63)
Any radiation therapy (vs. none)	134	36.7	1.04	(0.86, 1.25)	1.14	(0.91, 1.41)
Currently in treatment for initial cancer diagnosis	85	49.1	1.53	(1.26, 1.86)	1.30	(1.04, 1.63)
Months since diagnosis						
<18	142	39.1	1.00	ref	1.00	ref
18–23	21	34.4	0.88	(0.61, 1.27)	0.93	(0.63, 1.36)
24+	109	33.1	0.85	(0.69, 1.03)	0.98	(0.80, 1.20)

Abbreviations: PR, prevalence, ROCS, Research on Cancer Survivors, SD, standard deviation.

^a^ Adjusted models of survivor characteristics and decreased employment participation include sex, age, education, marital status, income, and occupation. Adjusted models of cancer characteristics and decreased employment participation control for survivor characteristics and cancer site, stage, treatments, treatment status, and time since diagnosis.

Decreased work participation was more common among lung and colorectal compared with breast cancer survivors. Distant stage (vs. localized) disease, receipt of chemotherapy, and being in active treatment at ROCS enrollment were associated with deceased work participation. Surgery, radiation, and time since diagnosis were not associated with decreased work participation.

Work‐related factors associated with decreased work participation are presented in Table 4. Taking at least one month of paid or unpaid time off was associated with decreased employment in a model controlling for demographic and cancer‐related factors. Paid time off was not associated with decreased work participation; however, decreased work participation was 29% (95% CI: 3%, 62%) more common among survivors who took any unpaid time off, and 77% (95% CI: 39%, 124%) more common among those who took at least one month of unpaid time off compared with those who did not. Changing work duties, schedules, and hours worked were associated with 69%, 54%, and 45% higher prevalence of decreased work participation, respectively.

**TABLE 4:** Prevalence ratios of time off and other work changes associated with decreased employment participation

	Decreased employment participation		Unadjusted		Adjusted for demographics^a^		Adjusted for demographics and cancer‐related factors^b^	
	N	%						
Took at least 1 month off (paid or unpaid)								
No	118	28.2	1.00	Ref	1.00	Ref	1.00	Ref
Yes	84	33.7	1.20	(0.95, 1.51)	1.46	(1.17, 1.83)	1.34	(1.06, 1.68)
Extended paid time off from work								
No	163	32.3	1.00	Ref	1.00	Ref	1.00	Ref
Yes	43	25.2	0.78	(0.58, 1.04)	1.04	(0.79, 1.36)	0.93	(0.71, 1.24)
1+ month sick time								
No	167	32.4	1.00	Ref	1.00	Ref	1.00	Ref
Yes	30	20.0	0.62	(0.44, 0.87)	0.87	(0.61, 1.23)	0.78	(0.54, 1.12)
1+ month vacation time								
No	184	30.8	1.00	Ref	1.00	Ref	1.00	Ref
Yes	16	23.9	0.75	(0.50, 1.21)	0.95	(0.64, 1.41)	0.84	(0.57, 1.26)
Any unpaid time off from work								
No	108	25.0	1.00	Ref	1.00	Ref	1.00	Ref
Yes	102	41.8	1.67	(1.34, 2.08)	1.37	(1.09, 1.72)	1.29	(1.03, 1.62)
1+ month unpaid time								
No	136	25.4	1.00	Ref	1.00	Ref	1.00	Ref
Yes	70	51.9	2.04	(1.64, 2.54)	1.88	(1.49, 2.38)	1.77	(1.39, 2.24)
Any work change								
No	77	21.9	1.00	Ref	1.00	Ref	1.00	Ref
Yes	137	39.0	1.78	(1.40, 2.27)	1.55	(1.21, 1.98)	1.37	(1.07, 1.75)
Changed work duties								
No	112	22.2	1.00	Ref	1.00	Ref	1.00	Ref
Yes	93	54.4	2.45	(1.98, 3.03)	1.80	(1.41, 2.30)	1.69	(1.33, 2.15)
Changed hours worked each week								
No	86	21.9	1.00	Ref	1.00	Ref	1.00	Ref
Yes	119	42.2	1.93	(1.53, 2.43)	1.63	(1.27, 2.07)	1.45	(1.14, 1.85)
Changed work schedule since diagnosis								
No	81	22.0	1.00	Ref	1.00	Ref	1.00	Ref
Yes	127	41.1	1.87	(1.48, 2.37)	1.70	(1.33, 2.16)	1.54	(1.21, 1.96)

Abbreviations: SD, standard deviation.

^a^ Models controlling for demographic factors include sex, age at diagnosis, education, marital status, household income, and occupation.

^b^ Models controlling for demographic and cancer‐related factors control for the demographic factors listed above, as well as for cancer site, stage, treatment types, treatment status, and time since diagnosis.

Sensitivity analyses including participants who reported being employed before their cancer diagnosis and retired at ROCS enrollment as having decreased employment are presented in Tables S1 and S2. These revealed similar patterns but weaker associations between individual and cancer‐related predictors of decreased work participation. Associations between time off work, work changes, and decreased work participation also attenuated when including retired survivors, suggesting that unpaid leave and work changes are more strongly associated with other work outcomes (going from full time to part time employment or from being employed to unemployed or on disability) than with retirement.

## 4 DISCUSSION

Work changes, including taking extended paid and unpaid time off and changing work schedules, duties, and hours worked were common in this population of African American cancer survivors. Nearly half of Detroit ROCS participants who were employed before diagnosis were not employed a median of 21 months after diagnosis. Post‐diagnosis employment status differed by whether survivors were employed full or part time before diagnosis, such that being on disability was more common among survivors employed full time before diagnosis while unemployment was more common among those employed part time. Cancer‐related predictors of decreased work participation include cancer site (lung or colorectal cancer), receipt of chemotherapy, and being in active treatment at ROCS enrollment. Work‐related predictors include taking unpaid time off and making work changes, including changing hours, duties, or schedules related to cancer. Together these results suggest that the burden of cancer on employment can last for months or years after diagnosis and that patients with fewer economic protections – including lower incomes and lack of access to paid time off work – may be at particular risk.

Our finding that 47.5% of ROCS participants decreased employment participation between diagnosis and study enrollment is in line with the findings of a systematic review of employment outcomes among cancer survivors that found that between 26% and 53% of cancer survivors lost their jobs or quit working in the 6‐year period after diagnosis.^28^ Close to half (46.5%) of employed ROCS participants made changes to their hours, schedules, or duties related to cancer, similar to estimates by Bradley, et al. that just over half (54.5%) of long‐term (5+ years) survivors of the same cancers reduced their schedules at some point because of cancer treatment,^10^ and results from MEPS estimating that 42.1% of survivors reported taking extended time off or making any change to their hours, duties, or employment status because of cancer.^29^

Our finding that just over one‐third of employed ROCS participants took extended paid time off is similar to an estimate from MEPS that 35.2% of employed survivors took extended paid time off; however, unpaid time off was much more common among ROCS participants than national estimates. While 33.7% of ROCS participants took unpaid time off, only 20.5% of MEPS participants took unpaid leave.^30^ Nearly one in five employed cancer survivors in ROCS took at least one month of unpaid time off, including 9% who reported taking 6 months or more of unpaid time (data not shown). Such extended unpaid absences from work put survivors at risk of income loss and other financial hardships, which are associated with clinically significantly lower levels of health‐related quality of life and, our results suggest, may be more common in African American survivors than among cancer survivors overall.^2^

Work outcomes are often worse among African American than white survivors.^12^, ^19^, ^20^, ^31^, ^32^ These differences may be due in part to differences in the types of jobs and their associated levels of compensation and employer‐sponsored benefits provided. Structural factors contribute to employment segregation such that African American adults are underrepresented in white collar and professional occupations, and are overrepresented in low‐paying jobs.^33^ In 2018, African Americans accounted for 12.3% of all employed persons ages 16 and older in the United States, but were underrepresented in management, professional, and related occupations (9.6%) and represented a larger proportion of the production, service, and transportation workforce (16.6%–17.3%).^13^ Previous work suggests that cancer survivors in blue collar occupations are less likely to retain jobs after cancer than those in white collar occupations^32^ and that reduced employment after cancer is associated with performing manual labor or having a physically demanding job.^15^, ^16^, ^17^, ^18^ In ROCS, decreased work participation was 57%–100% more common in craftspersons and those in operative or service‐related professions compared to survivors in professional or technical occupations. Occupation was no longer associated with decreased work participation in adjusted models, but these findings represent important differences in actual work outcomes among African American cancer survivors who are underrepresented in professional or technical fields with the most favorable employment outcomes for cancer survivors.

Differences in occupation type are associated with differences in compensation and employer‐sponsored benefits such as paid vacation and sick time and other employer accommodations that can help survivors retain and return to their jobs.^12^ Many of the business and professional occupations in which African American adults are underrepresented have average annual incomes of more than $75,000, compared to less than $40,000 for production, service, and transportation occupations.^14^ These occupational differences are also associated with different levels of employer‐provided benefits, such as paid sick leave and schedule flexibility that are associated with higher levels of job retention in cancer survivors.^28^, ^31^, ^32^ Among employed survivors in Detroit ROCS, taking extended paid sick time was associated with lower levels of decreased employment, although this association attenuated after controlling for income.

Household income information was available at ROCS enrollment but not before cancer diagnosis, and could have been impacted by employment changes. ROCS participants were separately asked whether their household income changed since their cancer diagnosis. In fully adjusted sensitivity analyses stratified by whether participants’ household incomes decreased since cancer diagnosis, lower household incomes were more strongly associated with decreased employment among survivors who did not experience a decrease in income (PR_$80,000+ vs. <$20,000_ 0.03, 95% CI: 0.007, 0.14) compared with those who did (PR_$80,000+ vs. <$20,000_ 0.59, 95% CI: 0.37, 0.95; *P*_interaction_ <0.001), suggesting that the observed association between lower income and decreased employment participation is not due to post‐diagnosis income changes.

Cancer site and sex likely both contribute to employment outcomes after cancer. Our findings suggest that decreased work participation was more common among lung and colorectal than breast or prostate cancer survivors. This may be due to differences in disease severity (in ROCS the majority of breast and prostate cancers were localized, while most lung and colorectal cancers were regional or distant at the time of diagnosis) and/or treatments received (receipt of chemotherapy was associated with decreased work participation, and treatment protocols differ among the sites included in ROCS, such that 60%–70% of breast, colorectal, and lung cancer survivors reported receiving chemotherapy compared with 4% of prostate cancer survivors). Because breast and prostate cancer survivors account for more than 75% of ROCS participants, we are unable to estimate differences in results by sex apart from differences in outcomes associated with individual cancer types; however, any future interventions developed to address employment and financial outcomes among cancer survivors should consider how cancer site and survivor sex may influence these outcomes.

In this study, we also found that post‐diagnosis employment status differed by whether survivors were working full or part time before diagnosis. In a systematic review of predictors of unemployment in breast cancer survivors, Wang, et al. reported no association between part time work and unemployment.^19^ This is consistent with our finding of similar levels of post‐diagnosis employment among survivors employed full‐ and part‐time before diagnosis; however, to the best of our knowledge, our finding of differences in post‐diagnosis disability and unemployment pre‐diagnosis employment status is novel and suggests that survivors working full time may have greater access to disability benefits and are at lower risk for cancer‐related unemployment than part‐time employees.

This study features many important strengths that contribute to its value in describing employment outcomes among employed cancer survivors. Detroit ROCS is a population‐based study using detailed surveys, including employment measures from a national study of the employment experiences of cancer survivors. The detailed data collected allowed for consideration of several cancer‐related, and work‐related factors as predictors of decreased work participation after cancer. By focusing on a population of African American survivors, this work describes employment outcomes among a population at higher risk for adverse financial and employment changes related to cancer, but that has been underrepresented in prior work.

This work should also be considered in the context of its limitations. While the inclusion of African American cancer survivors provides valuable insight into the employment outcomes of a survivor population that has been underrepresented in previous work, these findings may not be generalizable to other populations. Observed employment changes between cancer diagnosis and ROCS enrollment may not be attributable to cancer, and decreases in employment would not be captured for survivors who left work temporarily but returned before study enrollment. An average of 27 months elapsed between cancer diagnosis and ROCS enrollment, and longer time since diagnosis may be associated with inaccurate recall of employment impacts.

This work highlights disparities in employment outcomes after cancer such that more full‐time than part‐time employees have access to income replacement in the form of disability insurance, and more African American cancer survivors report taking unpaid time off as a result of cancer compared with nationally representative survivor samples. Efforts to increase access to paid time off and income replacement during and after cancer treatment may represent important strategies in reducing financial hardship experienced by African American cancer survivors and potentially to reduce disparities in financial hardship after cancer.

## CONFLICT OF INTEREST

Matthew P. Banegas received funding from AstraZeneca, paid to his institution, for an unrelated research project.

## Supporting information

Table S1‐S2

## Data Availability

The data that support the findings of this study are available from Detroit ROCS. Restrictions apply to the availability of these data, which were used with permission for this study. Detroit ROCS data may be requested at detroitrocs.org.
