# Supplementary material for: Work changes and individual, cancer‐related, and work‐related predictors of decreased work participation among African American cancer survivors
Source: Cancer Med. 2020 Nov 7;9(23):9168–77. doi: 10.1002/cam4.3512 (PMC7724298; doi:10.1002/cam4.3512)
Supplement: Supplementary file 1 — Table S1‐S2 [file CAM4-9-9168-s001.docx]

**Supplementary Table 1.** Decreased employment participation by participant sociodemographic, cancer-related, and work-related characteristics, including survivors who were retired at Detroit ROCS enrollment

|  | Decreased Employment Participation | | Unadjusted | | Adjusted | |
| --- | --- | --- | --- | --- | --- | --- |
|  | N | % | PR | 95% CI | PR | 95% CI |
| **Survivor characteristics** | 435 | 47.5 |  |  |  |  |
| Sex |  |  |  |  |  |  |
| Female | 223 | 46.2 | 1.00 | ref | 1.00 | ref |
| Male | 212 | 49.0 | 1.06 | (0.93, 1.22) | 1.08 | (0.85, 1.36) |
| Age at diagnosis |  |  |  |  |  |  |
| <55 | 130 | 35.3 | 1.00 | ref | 1.00 | ref |
| 55-64 | 209 | 52.6 | 1.49 | (1.26, 1.76) | 1.38 | (1.17, 1.62) |
| 65+ | 96 | 63.6 | 1.80 | (1.50, 2.16) | 1.72 | (1.41, 2.10) |
| Education |  |  |  |  |  |  |
| High school or below | 161 | 61.2 | 1.00 | ref | 1.00 | ref |
| Some college | 177 | 49.0 | 0.80 | (0.69, 0.92) | 0.95 | (0.82, 1.11) |
| College graduate | 89 | 31.9 | 0.52 | (0.43, 0.63) | 0.80 | (0.63, 1.01) |
| Marital status |  |  |  |  |  |  |
| Married/living with partner | 173 | 43.3 | 1.00 | ref | 1.00 | ref |
| Widowed | 39 | 70.9 | 1.64 | (1.34, 2.01) | 0.94 | (0.75, 1.18) |
| Divorced/separated | 116 | 50.4 | 1.17 | (0.98, 1.38) | 0.89 | (0.75, 1.06) |
| Never married | 105 | 46.5 | 1.07 | (0.90, 1.29) | 0.76 | (0.63, 0.92) |
| Annual household income |  |  |  |  |  |  |
| <$20,000 | 193 | 75.4 | 1.00 | ref | 1.00 | ref |
| $20,000-39,999 | 93 | 47.9 | 0.64 | (0.54, 0.75) | 0.66 | (0.56, 0.78) |
| $40,000-79,999 | 78 | 244 | 0.42 | (0.35, 0.52) | 0.44 | (0.36, 0.54) |
| $80,000+ | 35 | 21.5 | 0.28 | (0.21, 0.39) | 0.30 | (0.21, 0.41) |
| Usual occupation |  |  |  |  |  |  |
| Professional/technical | 101 | 39.5 | 1.00 | ref | 1.00 | ref |
| Manager/administrator | 43 | 39.5 | 1.00 | (0.76, 1.32) | 0.97 | (0.76, 1.23) |
| Clerical | 29 | 40.3 | 1.02 | (0.74, 1.41) | 0.75 | (0.52, 1.08) |
| Service | 86 | 59.3 | 1.50 | (1.23, 1.84) | 0.84 | (0.67, 1.05) |
| Craftsperson | 42 | 54.6 | 1.38 | (1.07, 1.78) | 0.86 | (0.65, 1.14) |
| Operative | 87 | 50.9 | 1.29 | (1.04, 1.59) | 0.88 | (0.70, 1.11) |
| **Cancer-related factors** |  |  |  |  |  |  |
| Site |  |  |  |  |  |  |
| Breast | 152 | 41.0 | 1.00 | ref | 1.00 | ref |
| Colorectal | 78 | 59.1 | 1.44 | (1.20, 1.74) | 1.27 | (0.98, 1.65) |
| Lung | 57 | 79.2 | 1.93 | (1.63, 2.29) | 1.36 | (1.06, 1.76) |
| Prostate | 148 | 43.3 | 1.06 | (0.89, 1.26) | 1.07 | (0.78, 1.46) |
| Stage |  |  |  |  |  |  |
| Localized | 228 | 41.3 | 1.00 | ref | 1.00 | ref |
| Regional | 146 | 51.4 | 1.24 | (1.07, 1.45) | 1.03 | (0.88, 1.21) |
| Distant | 59 | 77.6 | 1.88 | (1.61, 2.20) | 1.24 | (1.00, 1.55) |
| Treatment |  |  |  |  |  |  |
| Any surgery (vs. none) | 310 | 45.7 | 0.86 | (0.75, 1.00) | 1.06 | (0.90, 1.25) |
| Any chemotherapy (vs. none) | 207 | 53.8 | 1.25 | (1.10, 1.44) | 1.20 | (1.00, 1.44) |
| Any radiation therapy (vs. none) | 216 | 48.3 | 1.04 | (0.91, 1.19) | 1.07 | (0.91, 1.25) |
| Currently in treatment for initial cancer diagnosis | 121 | 57.9 | 1.30 | (1.13, 1.51) | 1.18 | (1.00, 1.39) |
| Months since diagnosis |  |  |  |  |  |  |
| <18 | 189 | 46.1 | 1.00 | ref | 1.00 | ref |
| 18-23 | 35 | 46.7 | 1.01 | (0.78, 1.32) | 1.06 | (0.80, 1.41) |
| 24+ | 211 | 49.0 | 1.06 | (0.92, 1.22) | 1.13 | (0.97, 1.31) |

Abbreviations: PR - prevalence, SD – standard deviation.

^a^ Adjusted models of survivor characteristics include sex, age, education, marital status, income, and occupation. Adjusted models of cancer characteristics control for survivor characteristics and cancer site, stage, treatments, treatment status, and time since diagnosis.

**Supplementary Table 2:** Prevalence ratios of time off and other work changes associated with decreased employment participation.

|  | Decreased employment participation | |  | | Adjusted for demographics^a^ | | Adjusted for demographics and cancer-related factors^b^ | |
| --- | --- | --- | --- | --- | --- | --- | --- | --- |
|  | N | % | Unadjusted | |  |  |  |  |
| **Took at least 1 month off (paid or unpaid)** |  |  |  |  |  |  |  |  |
| No | 201 | 40.0 | 1.00 | ref | 1.00 | ref | 1.00 | ref |
| Yes | 101 | 38.0 | 0.95 | (0.79, 1.14) | 1.24 | (1.03, 1.49) | 1.17 | (0.97, 1.41) |
| **Extended paid time off from work** |  |  |  |  |  |  |  |  |
| No | 252 | 42.4 | 1.00 | ref | 1.00 | ref | 1.00 | ref |
| Yes | 56 | 30.4 | 0.72 | (0.57, 0.91) | 0.98 | (0.77, 1.24) | 0.91 | (0.72, 1.16) |
| 1+ month sick time |  |  |  |  |  |  |  |  |
| No | 256 | 42.4 | 1.00 | ref | 1.00 | ref | 1.00 | ref |
| Yes | 41 | 25.5 | 0.60 | (0.45, 0.80) | 0.86 | (0.64, 1.14) | 0.79 | (0.59, 1.06) |
| 1+ month vacation time |  |  |  |  |  |  |  |  |
| No | 282 | 40.6 | 1.00 | ref | 1.00 | ref | 1.00 | ref |
| Yes | 17 | 25.0 | 0.62 | (0.40, 0.94) | 0.84 | (0.57, 1.24) | 0.79 | (0.54, 1.15) |
| **Any unpaid time off from work** |  |  |  |  |  |  |  |  |
| No | 192 | 37.2 | 1.00 | ref | 1.00 | ref | 1.00 | ref |
| Yes | 120 | 45.8 | 1.23 | (1.04, 1.46) | 1.21 | (1.01, 1.45) | 1.14 | (0.95, 1.36) |
| 1+ month unpaid time |  |  |  |  |  |  |  |  |
| No | 229 | 36.4 | 1.00 | ref | 1.00 | ref | 1.00 | ref |
| Yes | 77 | 54.2 | 1.49 | (1.24, 1.79) | 1.55 | (1.28, 1.89) | 1.47 | (1.21, 1.78) |
| **Any work change** |  |  |  |  |  |  |  |  |
| No | 145 | 36.1 | 1.00 | ref | 1.00 | ref | 1.00 | ref |
| Yes | 165 | 43.5 | 1.21 | (1.01, 1.44) | 1.17 | (0.98, 1.40) | 1.04 | (0.87, 1.25) |
| Changed work duties |  |  |  |  |  |  |  |  |
| No | 197 | 33.5 | 1.00 | ref | 1.00 | ref | 1.00 | ref |
| Yes | 106 | 57.6 | 1.72 | (1.46, 2.04) | 1.51 | (1.25, 1.82) | 1.47 | (1.22, 1.78) |
| Changed hours worked each week |  |  |  |  |  |  |  |  |
| No | 170 | 35.6 | 1.00 | ref | 1.00 | ref | 1.00 | ref |
| Yes | 134 | 45.1 | 1.27 | (1.06, 1.51) | 1.25 | (1.04, 1.50) | 1.16 | (0.97, 1.40) |
| Changed work schedule since diagnosis |  |  |  |  |  |  |  |  |
| No | 156 | 35.1 | 1.00 | ref | 1.00 | ref | 1.00 | ref |
| Yes | 153 | 45.7 | 1.30 | (1.09, 1.54) | 1.29 | (1.08, 1.53) | 1.17 | (0.98, 1.39) |

Abbreviations: SD – standard deviation.

^a^ Models controlling for demographic factors include sex, age at diagnosis, education, marital status, household income, and occupation.

^b^ Models controlling for demographic and cancer-related factors control for the demographic factors listed above, as well as for cancer site, stage, treatment types, treatment status, and time since diagnosis.
